# Metachronous colorectal carcinoma with massive submucosal invasion detected by annual surveillance in a Lynch syndrome patient: a case report

**DOI:** 10.1186/s12957-017-1207-3

**Published:** 2017-08-01

**Authors:** Masashi Utsumi, Kohji Tanakaya, Yutaka Mushiake, Tomoyoshi Kunitomo, Isao Yasuhara, Fumitaka Taniguchi, Takashi Arata, Koh Katsuda, Hideki Aoki, Hitoshi Takeuchi

**Affiliations:** Department of Surgery, National Hospital Organization, Iwakuni Clinical Center, 1-1-1 Atago-machi, Iwakuni-shi, Yamaguchi, 740-8510 Japan

**Keywords:** Colorectal carcinoma, Lynch syndrome, Surveillance, Colonoscopy, Laparotomy

## Abstract

**Background:**

Lynch syndrome is the most common form of hereditary colorectal carcinoma. It is characterized by the presence of germline mutations in DNA mismatch repair genes. Mutation carriers have a lifetime risk of developing colorectal carcinoma of approximately 80%. Current treatment guidelines recommend periodic surveillance for colorectal carcinoma in patients with Lynch syndrome. However, the optimal interval between colonoscopies has not yet been determined.

**Case presentation:**

We describe a 54-year-old man with Lynch syndrome who was undergoing annual colonoscopy surveillance for the development of colorectal carcinoma. At 54, 57, 59, and 60 years old, a colonoscopy showed high-grade dysplasia and adenoma. Therefore, endoscopic mucosal resection was performed. At 61 years old, a colonoscopy showed metachronous colorectal carcinoma with massive submucosal invasion. He subsequently underwent laparotomy for colorectal carcinoma.

**Conclusions:**

Annual surveillance using colonoscopy can detect colorectal carcinoma at an early stage, leading to reduced mortality. However, some patients might require a laparotomy, as was the case here. More frequent colonoscopic surveillance might be necessary to avoid surgery for colorectal carcinoma in Lynch syndrome patients with multiple risk factors for interval cancer.

## Background

Lynch syndrome is the most common form of hereditary colorectal carcinoma (CRC), accounting for 2–4% of all colorectal and endometrial cancers [1]. It is defined as the presence of germline mutations in DNA mismatch repair (MMR) genes including *MSH2*, *MLH1*, *MSH6*, and *PMS2* [2]. The syndrome is characterized by the development of CRC and various other cancers that are frequently diagnosed at an early age [3]. The lifetime cumulative CRC risk is estimated to be as high as 50–80% [4]. Early-stage CRC can be detected by colorectal surveillance, resulting in early treatment and reduced mortality [5]. Current surveillance guidelines recommend an interval of 1–2 years between colonoscopies [3]. Physicians face a range of difficult decisions regarding prophylaxis and surveillance. Prophylactic colectomy is a possible treatment option, but colonoscopic surveillance, which is safe and effective, is the favored approach [6]. Because adenoma is thought to be the precursor to CRC in mutation carriers, guidelines recommend colonoscopic surveillance in patients with Lynch syndrome. Current surveillance guideline recommendations are based partly on data suggesting that the adenocarcinoma sequence is accelerated in patients with Lynch syndrome [3, 7].

In the present case, we describe a man with Lynch syndrome who presented with metachronous CRC with massive submucosal invasion and who underwent laparotomy, a year after an annual surveillance colonoscopy.

## Case presentation

**Fig. 1:**
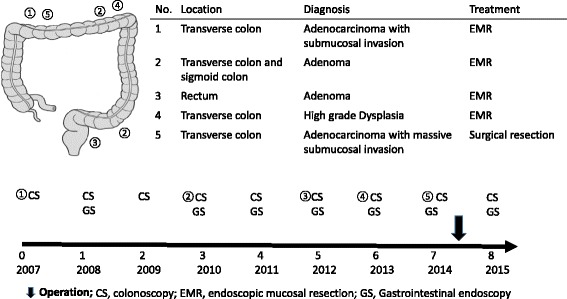
Clinical course from the first colonoscopy to the laparoscopic-assisted subtotal colectomy with lymphadenectomy

In September 2006, a 54-year-old man visited our hospital for genetic counseling because his cousin had Lynch syndrome. His past medical history was not significant; he stopped using tobacco when he was 30 years old and was an infrequent alcohol drinker. He reported an overwhelming family history, which fulfilled the criteria that enable the selection of families that are at risk for Lynch syndrome [8]. After obtaining informed consent, genetic testing for MMR mutations identified a germline mutation in *MLH1*, Exon 5 c.381-431_c.453+717del1221. Therefore, surveillance for Lynch syndrome was initiated at our institution in 2007. The clinical course of the patient is shown in Fig. 1.

**Fig. 2:**
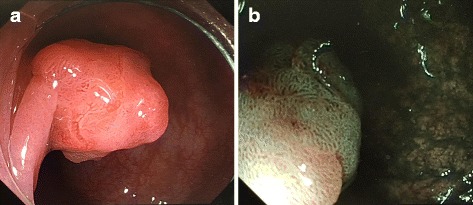
**a** Light standard colonoscopy. Colonoscopy revealed an elevated 2-cm diameter lesion located 70 cm from the anal verge in the transverse colon. **b** Narrow Band Imaging (NBI). The tumor showed an NBI C2/C3, CV Vit-H irregular image

At 54 years old, colonoscopy showed an elevated lesion in the transverse colon. Biopsy confirmed the lesion was adenocarcinoma. We performed an endoscopic mucosal resection (EMR). Histological examination revealed the tumor to be a well-differentiated adenocarcinoma that invaded the submucosal layer. At 57 years old, colonoscopy showed two elevated lesions in the transverse and sigmoid colon, and EMR was performed on these lesions. Histological examination revealed those tumors to be an adenoma. At 59 years old, colonoscopy showed an elevated 3-mm lesion in the rectum. We performed EMR on the lesion, which was also histologically confirmed as an adenoma. At 60 years old, colonoscopy showed an elevated 10-mm diameter lesion in the transverse colon. We performed EMR, and the lesion was histologically diagnosed as high-grade dysplasia. The adenomas resected at 57 and 60 years old were flat adenomas. At 61 years old, colonoscopy revealed an elevated 2-cm diameter lesion located 70 cm from the anal verge in the transverse colon. The tumor was evaluated using narrow band imaging (NBI) C2/C3, CV Vit-H irregular image [9], and massive invasive cancer was suspected (Fig. 2). According to the endoscopic findings, the tumor could not be resected en bloc by endoscopic submucosal dissection. The lesion was diagnosed as CRC invading the massive submucosal layer, and the patient underwent a laparoscopic-assisted subtotal colectomy with lymphadenectomy. The postoperative course was uneventful, and the patient was discharged without defecation problems on postoperative day 12. The resection specimen contained a 2-cm, moderately differentiated adenocarcinoma. Histological examination revealed that the tumor invaded the submucosal and vascular layers, which is an indication for intestinal resection with lymph node dissection [10]. Total 25 lymph nodes were removed. Lymph nodes were negligible, resulting in a classification of T1N0M0 (stage I: Union for International Cancer Control Tumor-Node-Metastasis staging).

**Fig. 3:**
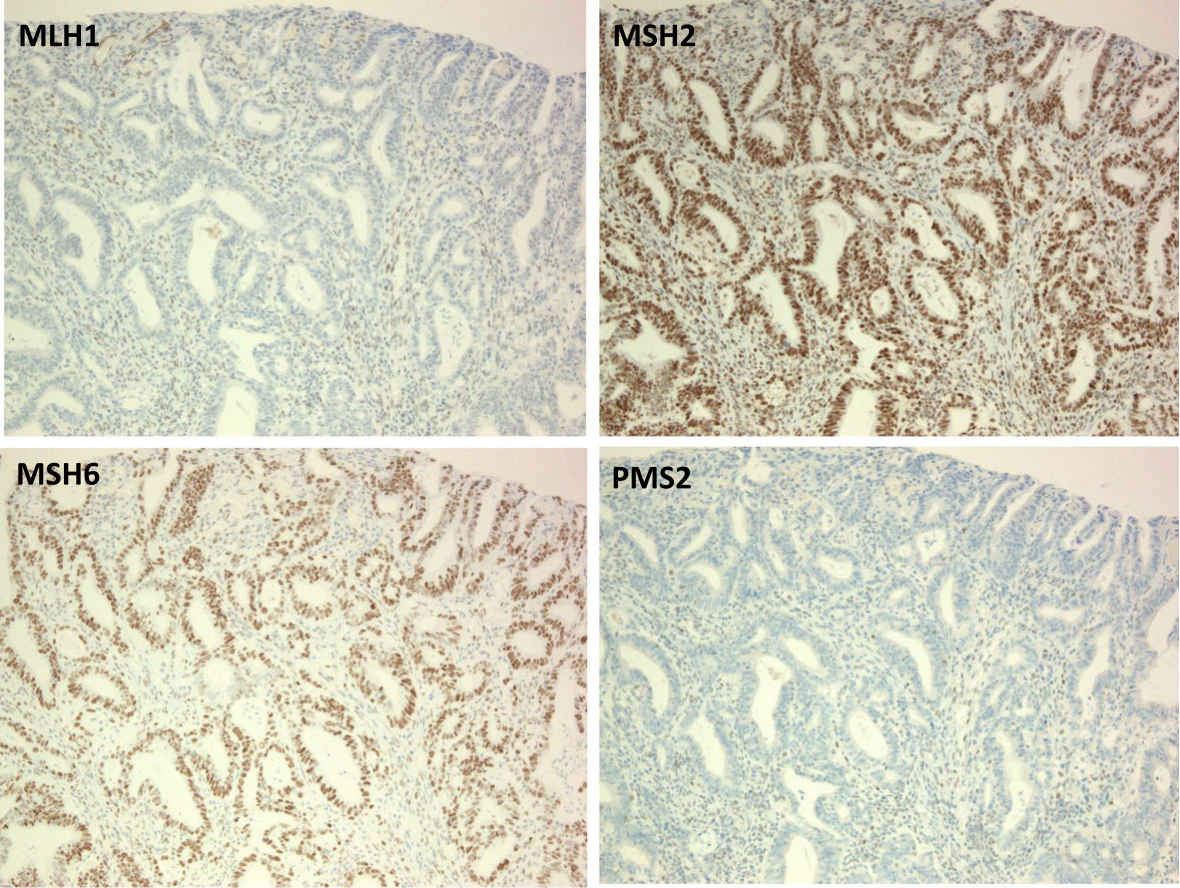
Immunohistochemical staining of the colon cancer. The colon cancer stained negative for MLH1 and PMS2 and positive for MSH6 and MSH2 (×400), which was in agreement with the germline mutation in *MLH1*

Immunohistochemical staining demonstrated that the tumor was positive for MSH2 and MSH6 and negative for MLH1 and PMS2 (Fig. 3), which was in agreement with the germline mutation in *MLH1*. A microsatellite instability analysis using the National Cancer Institute panel revealed that the tumor had high microsatellite instability. These results demonstrated that the tumor was associated with Lynch syndrome. The findings on postoperative follow-up and surveillance testing a year later were unremarkable.

## Discussion

Colorectal adenoma is considered a premalignant lesion [11]*.* Compared with the sporadic adenoma in the general population, the clinical characteristics of adenoma in Lynch syndrome include an earlier onset (by age 40 years), higher nuclear grade, and faster progression to cancer (within 5 years) [12]. Kalady et al. reported that the lifetime cumulative number of colorectal adenomas was about 20 [13]. It was reported that in Lynch syndrome surveillance, polypectomy of adenoma and early detection of cancer by colonoscopic surveillance reduce the risk of CRC development and mortality. All adenomas are indicators for resection, regardless of size [14].

In sporadic cancer, indication criteria for endoscopic resection include high-grade dysplasias or carcinomas with slight submucosal invasion of any size or macroscopic type [10]. Furthermore indication criteria for additional surgical treatment after endoscopic resection of carcinoma with submucosal invasion include a positive ventral margin or any of the following histological features: (1) depth of SM invasion ≥1000 μm; (2) vascular invasion; (3) poorly differentiated adenocarcinoma, signet-ring cell carcinoma, or mucinous carcinoma; and (4) grade 2/3 budding at the deepest invasion site [10]. In the present case, the tumor invaded the vasculature; therefore, an endoscopic resection was not indicated.

Järvinen et al. reported that colonoscopic screening at 3-year intervals reduced the incidence rate of CRC by 62% and decreased the overall mortality by approximately 65% [5]. Because interval cancer is detected between 2 and 3 years after surveillance colonoscopy [15] and almost all CRCs develop after 20 years of age, colonoscopic surveillance should be performed at least every 2 years, beginning between the ages of 20 and 25 years [14]. Vasen et al. [16] indicated that for carriers of *MLH1* or *MSH2* mutations, a surveillance interval of 1–2 years reduced the risk of CRC compared with one of 2–3 years. Engel et al. indicated the efficacy of annual colonoscopic surveillance [17] . We adopted the recommended routine surveillance protocol for Lynch syndrome [3]. The cumulative risk of metachronous CRC 10 years after segmental colectomy was suggested to be 16–19% [18, 19]. However, there is scant evidence for the age to initiate screening, and the debate regarding the optimal surveillance timing continues, suggesting that there is room for improvement in surveillance methods.

Interval cancer risk factors include being a carrier >40 years of age, an *MLH1* or *MSH2* mutation carrier, an incomplete previous endoscopy, residual adenomatous tissue, and flat adenomas [20]. There are limited data regarding whether new endoscopic techniques, such as intensive colonoscopy [21], chromoendoscopy [22], narrow-band imaging [23], or autofluorescence endoscopy [24] are superior to white light standard colonoscopy for polyp detection in Lynch syndrome.

In the present case, the patient was at high risk for CRC because he was a carrier >40 years of age, a carrier of an *MLH1* gene mutation, and had a history of EMR for CRC invading the submucosal layer and flat adenomas. As expected, he developed metachronous CRC and underwent laparotomy during intensive surveillance, as recommended in the guidelines. However, if more intensive surveillance was conducted, laparotomy might have been avoided.

As this is a single case report, further research is needed to draw definitive conclusions regarding the utility of colonoscopy at surveillance intervals shorter than a year; nevertheless, the present case indicates that such examinations are warranted. In addition, intensive surveillance might improve patient prognosis. Moreover, in the absence of a randomized controlled trial or comparative observation study, it is difficult to provide specific recommendations on the interval.

## Conculsions

﻿Annual surveillance by colonoscopy can detect CRC at an early stage, leading to reduced mortality. However, some patients might require a laparotomy even when under annual surveillance, as was the case here. More frequent colonoscopic surveillance at intervals less than 1 year might be necessary to avoid CRC surgery in Lynch syndrome patients with multiple risk factors for interval cancer. In conclusion, the present case suggests that colonoscopy at surveillance intervals shorter than a year might be useful for the early detection of CRC.

## Abbreviations

CRC: Colorectal cancer
EMR: Endoscopic mucosal resection
MLH1: MutL homolog 1
MMR: Mismatch repair
MSH2: MutS homolog 2
MSH6: MutS homolog 6
PMS2: PMS1 homolog 2, mismatch repair system component
